# Influence of Turbulence, Orientation, and Site Configuration on the Response of Buildings to Extreme Wind

**DOI:** 10.1155/2014/178465

**Published:** 2014-02-19

**Authors:** Aly Mousaad Aly

**Affiliations:** Department of Civil and Environmental Engineering, Louisiana State University, 3513D Patrick Franck Taylor Hall, Baton Rouge, LA 70803, USA

## Abstract

Atmospheric turbulence results from the vertical movement of air, together with flow disturbances around surface obstacles which make low- and moderate-level winds extremely irregular. Recent advancements in wind engineering have led to the construction of new facilities for testing residential homes at relatively high Reynolds numbers. However, the generation of a fully developed turbulence in these facilities is challenging. The author proposed techniques for the testing of residential buildings and architectural features in flows that lack fully developed turbulence. While these methods are effective for small structures, the extension of the approach for large and flexible structures is not possible yet. The purpose of this study is to investigate the role of turbulence in the response of tall buildings to extreme winds. In addition, the paper presents a detailed analysis to investigate the influence of upstream terrain conditions, wind direction angle (orientation), and the interference effect from the surrounding on the response of high-rise buildings. The methodology presented can be followed to help decision makers to choose among innovative solutions like aerodynamic mitigation, structural member size adjustment, and/or damping enhancement, with an objective to improve the resiliency and the serviceability of buildings.

## 1. Introduction

### 1.1. Background

Wind can be low, moderate, strong, and extremely destructive. While low and moderate winds are beneficial for pollution dispersion and electric power generation, strong and extreme wind events can cause devastating effects on the infrastructure. Extreme winds may cause damage to low-rise buildings in the form of windows damage, roof loss, or even complete collapse of wooden structures. In tall buildings, however, both cladding loads and the dynamics of the structure become a concern. Wind-induced response/loading in structures depend on, among other factors, (1) terrain or mean wind velocity profile and turbulence characteristics, (2) building's aerodynamic shape, (3) wind speed (which should not always be very high to cause damage, for instance, Tacoma Narrows bridge failed under moderate wind speed (about 40 mph), however, negative aerodynamic damping (self-excitation) existed [1]), (4) wind direction, and (5) structural properties that may magnify wind loads at resonance. The use of high-strength, lightweight materials, longer floor spans, and more flexible framing systems results in buildings that are more prone to vibrations. Wind events can cause severe and/or sustained vibratory motion in high-rise buildings, which can be detrimental to both the structure and occupants. Wind-induced vibrations may cause structural damage, annoyance to the occupants (especially in the upper floors), and/or impaired function of instruments. The evaluation of wind-induced loads and responses is an important step for the design of the main force-resisting system of high-rise buildings, to balance safety and serviceability issues with the reality of limited resources.

### 1.2. Response of Buildings to Extreme Winds

Wind-induced response in high-rise buildings, in the along-wind direction, may be evaluated using formulae provided in the literature [2–6]. However, the literature has less than comprehensive information for the evaluation of the critical cross-wind and torsional responses. It is difficult to have an acceptable direct analytical relation for the evaluation of the cross-wind and torsional response from the oncoming flow fluctuations [7, 8]. In addition, the interference effects of surrounding tall buildings represent another challenge. Moreover, the responses evaluated using those formulae are restricted to a few modes, and the process depends on a lot of assumption. On the other hand, wind-induced pressure measurements and finite element (FE) modeling of structures are an effective alternative for determining these responses [9, 10].

Wind tunnel tests have been commonly used as reliable methods for estimating wind loads on high-rise buildings. There are two types of rigid model testing that can provide overall structural wind loads. One method relay on high-frequency base balance (HFBB) measurements and the second method is based on high-frequency pressure integration (HFPI) of loads. The HFBB approach can only provide global wind loads at the base of the test model. The test results from the HFBB measurements can be analyzed using frequency-domain or time-domain techniques to obtain the response of a building. The frequency domain approach has been dominant over time-domain approach for its lesser requirement of computational power though it involves more approximations compared to the time-domain approach. Nevertheless, with the current technology where computational power has been significantly improved, the time-domain method will become a popular analysis technique. The time-domain method allows the determination of wind responses directly from the equation of motion using the measured time history of wind loads, thereby avoiding all the simplifying assumptions used in the frequency domain method. However, even if the accurate time-domain approach is used for the analysis of the response, the three-dimensional (3D) mode shapes found in complex tall buildings complicate the use of the HFBB results for predicting the structural response [11, 12]. In general, mode shape correction factors for the HFBB technique are necessary for the assessment of wind-induced responses of a tall building. This is to account for the significant uncertainties in the estimation of generalized forces due to the nonideal mode shapes as well as presumed wind loading distributions [13, 14]. The HFPI technique with the time-domain approach can be more accurate, provided that enough coverage of pressure taps on the model's outer surface is considered [9, 10, 15–19].

The HFPI technique is based on simultaneous pressure measurements at several locations on a building's outer surface. Pressure data can be used for the design of the claddings as well as the estimation of the overall design loads for the main force resisting system. The HFPI technique cancels out any inertial effects that may be included in the overall loads measured by the base balance when the HFBB technique is used. Time histories of wind forces at several levels of tall building models can be obtained from a boundary-layer wind tunnel experiment, with a multichannel pressure scanning system. This enables the building responses to be computed directly in the time domain for buildings with simple or complex mode shapes [9, 10, 19, 20]. A part from the methodology used for response estimation in buildings, turbulence, and the interference from the surrounding plays an important role in the dynamic behavior of buildings under wind.

### 1.3. The Role of Turbulence

Atmospheric turbulence results from the vertical movement of air, together with flow disturbances around surface obstacles which makes low- and moderate-level winds extremely irregular [21]. In environmental sciences, turbulence is important because it mixes the atmosphere causing energy, water vapor, smoke, pollutants, and other substances to become distributed widely. In wind engineering, turbulence influences the dynamic response and the total wind loading on buildings and other structures. To understand how turbulence affects a building, it should be first either physically or numerically modelled to permit the evaluation of its effects. In 1941, Kolmogorov attempted to model turbulence in the atmosphere using a statistical approach [22, 23]. Even with advancements in computational fluid dynamics (CFD), time history simulations of turbulent flows on large structures is an extremely time and resource consuming task. Accordingly, laboratory testing may be a feasible approach by which the complex wind-induced loads on a structure can be obtained in a relatively short time, provided that the simulated turbulence is fully developed. Nevertheless, the simulation of turbulence at modern wind engineering testing facilities is challenging [24], which raises the question: *to what extent should turbulence be accurately simulated in a laboratory?*

Lee [25] studied the effects of turbulence scale on mean forces on square prisms. The results indicated that care must be taken when modeling a building in natural wind to ensure that ratios of turbulence scale size to building dimensions remain constant. In addition, at high values of turbulence scale, the drag tends to have a large constant value compared to low turbulence scales. The importance of the similarity of the integral length scale of turbulence and the test model geometric scale is stressed in Cook [26] as follows: “owing to the functional dependence of *L*_*ux*_ on *z*_0_ and (*z* − *d*), a model boundary-layer will have a unique scale factor. The linear scale of any building model should be matched to this scale factor; otherwise the scales of the simulated atmospheric turbulence and the building-generated turbulence will not match. In that event, the scaled dynamic response of the model in load or in deflection will not be correct.” Research carried out by Holdo et al. [27] suggests that length scales are very important scaling parameters and should be maintained in testing of low-rise buildings.

Laneville [28] presented a review of turbulence scale effect on mean drags of two dimensional rectangular cylinders. The review shows a possible turbulence scale effect on the mean drag as the turbulence scale ratio, *L*_*ux*_/*D*, is larger than three. The order of magnitude of this effect is about 25% and 12% on the mean base pressure and mean drag coefficients, respectively, for *L*_*ux*_/*D* of about twenty. Wang et al. [29] carried out a pressure distribution study on a building model in wind. They concluded that, as long as the roughness height is correctly modeled (i.e., turbulence intensity and mean velocity profiles are properly simulated), accurate results of pressure coefficients on the building surface can be obtained. This result is also valid even if the scale ratio of the body size to the integral length scale is not correctly selected. Dalley [30] presented surface pressure investigations on a model of the Silsoe structures building, along with a comparison with full-scale data. The study showed differences between model and full-scale pressure coefficients, which may be related to discrepancies in the approaching flow. Tieleman et al. [31] compared wind tunnel pressure measurements with full-scale experimental data for a low-rise building. The results showed that pressure coefficients on a full-scale experimental building are best duplicated by a wind tunnel simulation which reproduces turbulence intensities and small-scale turbulence content in the incident flow. However, exact scaling of the turbulence integral length scale in a wind tunnel experiment does not seem to be essential for the prediction of wind loads on low-rise buildings. Even when the integral length scale is well simulated in a wind tunnel, full-scale studies at Silsoe cast doubt on the basic assumption that bluff-body flows are insensitive to Reynolds number. However, the implication for high-rise buildings is likely to be more significant than for low-rise buildings [32].

According to Richards et al. [33], at relatively large-scale wind-tunnel modeling of civil engineering structures, it is very difficult to model the full turbulence spectra and so only the high-frequency end of the spectrum is matched. Bienkiewicz et al. [34] carried out a comparative study of approach flow and wind pressures on low-rise buildings using wind tunnel data generated at six wind engineering laboratories. They investigated the variability in the laboratory wind loading [4, 35]. The comparative results (as presented in [35]) show large variability in the measured wind loads. This variability was primarily attributed to differences in the approach flows employed in the physical modeling of wind pressures on tested buildings. The variability in the approach flows resulted, to a large extent, from the differences in the along-wind turbulence intensity implied by different empirical models. This is further complicated when involving the dynamic behavior of a high-rise building.

Mathematical modelling of turbulence in CFD requires relatively long simulation time and potential computational resources. Since the 1960's wind tunnels have been enjoying a renewed interest as a means by which wind loads can be predicted in a relatively short time. Recently, open-jet facilities are used for the modelling of the atmospheric flows on buildings [24]. However, the generation of a fully developed turbulent flow in such facilities is challenging. Research carried out by the author in Fu et al. [36] and Aly and Bitsuamlak [37] suggests that wind loads on low-rise buildings and small-scale structures can be fairly estimated in flows that lack low-frequency turbulence (large eddies). However, such an approach may not be fully applicable for large and flexible structures.

### 1.4. Paper Layout

This paper presents a detailed study to investigate the influence of upstream turbulence, wind direction (orientation), and the interference from the surrounding on the responses of tall buildings in the time domain. In the theoretical formulation, dynamic equations are introduced by FE analysis; and then a solution procedure for a set of ordinary differential equations is considered. The paper is organized as follows.  Section 2 presents the methodology followed in this paper which consists of wind tunnel testing, FE modelling, and reproduction of wind loads at full-scale. The response obtained using the wind tunnel data and the FE model of a tall building are presented in Section 3. In Section 4, a discussion of the results with further explanation of the vortex shedding mechanism and typical mitigation techniques for response reduction are presented.  Section 5 presents the conclusions drawn from the current study along with recommendations for future work.

## 2. Methodology

An FE three-step analysis procedure based on wind tunnel pressure measurements was followed to obtain the response of a tall building to wind. The proposed methodology has the advantages of combining lateral along-wind, lateral cross-wind, and torsional responses altogether. The proposed technique has the capabilities of considering structures with (1) complex mode shapes, (2) nonuniform mass distribution, (3) higher modes of vibration, (4) flow variability, and (5) interference from the surrounding at any wind direction angle [10].

### 2.1. Experimental Testing

The first step in the proposed procedure is to obtain time histories of actual wind loads on a tall building. This was achieved by a wind tunnel experiment on a high-rise building model which was carried out at the PoliMI wind tunnel, Milano, Italy [9, 19]. Pressure measurements were conducted using high speed PSI-system 8400. Overall base loads were obtained by a force balance and compared with total loads attained from the integration of the pressures to ascertain sufficient tap coverage. The dimensions of the boundary-layer test section were 4 m height, 14 m width, and 36 m length. These huge dimensions allow for testing civil engineering structural models at large scales (up to 1 : 50) with low blockage effects. The long length of the test section allows for the generation of a fully developed turbulence. The empty test section provides a very uniform smooth flow with a boundary-layer thickness of about 0.2 m and a turbulence intensity *I*_*u*_ below 2%, due to a special type of painting used for floor and walls. Passive vortex generators in the form of spires, bricks, and roughness elements in the shape of pyramids were used at the entrance of the test section to simulate the growth of the boundary-layer.

**Figure 1:**
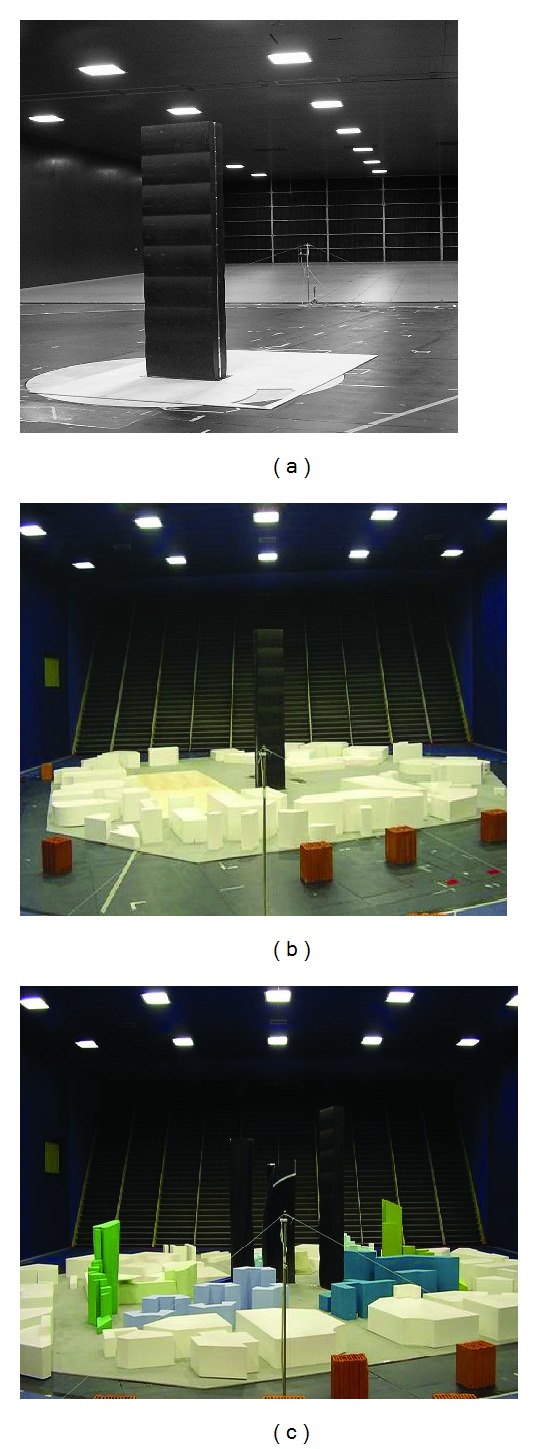
Wind tunnel configurations: (a) configuration SF (table configuration is the same for BLa and BLb); (b) table configuration I (for a wind direction angle of −67.5°); (c) table configuration II (for a wind direction angle of 22.5°).

**Figure 2:**
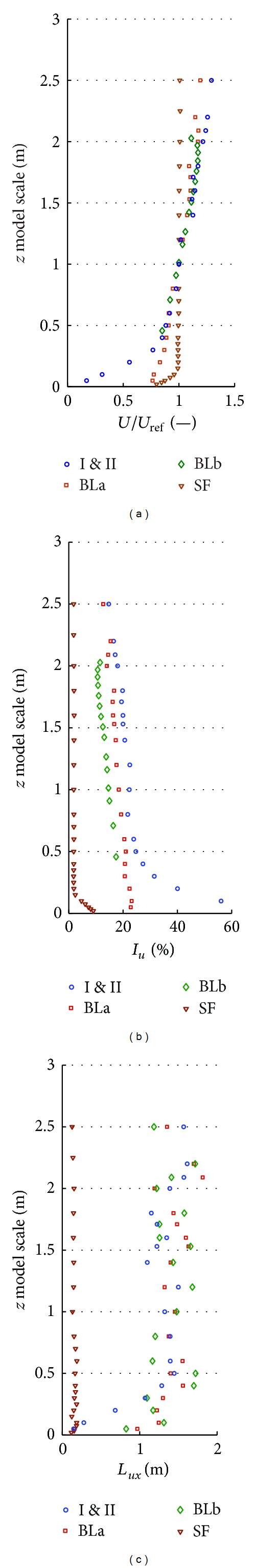
Flow characteristics: (a) mean wind velocity profiles; (b) turbulence intensity profiles; (c) integral length scales of turbulence.

**Figure 3:**
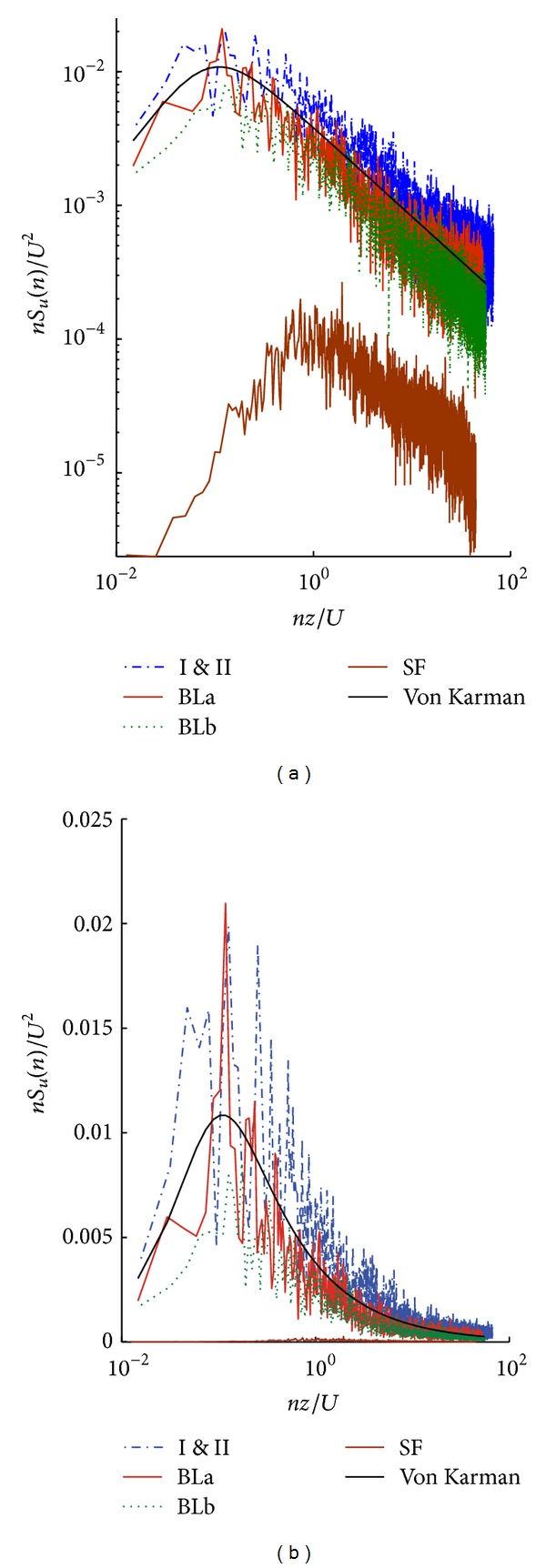
Spectra of the along-wind velocity components for different flow conditions with the von Karman spectrum: (a) logarithmic scale; (b) linear scale of the *y*-axis. While the flow in configuration BLb is slightly missing turbulence, especially at low-frequencies, the smooth flow is missing both large and small eddies (low- and high-frequency turbulence, resp.).

Five testing configurations were considered in this study.  Figure 1 shows photographs of the site configurations used. Site configurations I and II (Figures 1(b) and 1(c)) have the wind profiles indicated in Figure 2 [18, 38]. Configurations SF, BLa, and BLb have the same site configuration as in Figure 1(a) but different profiles as indicated in Figure 2. The main characteristics of the flows in the test section are given in Table 1.  Figure 3 shows spectra of the along-wind velocity components for different flow conditions plotted with the von Karman spectrum. While the flow in configuration BLb is slightly missing turbulence, especially at low-frequencies, the smooth flow is missing both large and small eddies (low- and high-frequency turbulence, resp.).

The building used in this study is 209 m × 57.6 m × 22 m (*B*/*D* = 2.6; *B*: chord length, *D*: thickness) steel tower with a total weight of 4 × 10^7^ kg. The aspect ratio is about 9.5, which makes it very sensitive to strong winds. A rigid model of the tower made of carbon fiber and geometrically scaled 1 : 100 was used in the experimental testing. The surrounding buildings within a radius of 500 m from the center of the main test building were scaled 1 : 100 and presented on a turn-table according to the configuration used. Pressure data were collected by a total of 448 taps at a sample rate of 62.5 Hz.

### 2.2. FE Modeling

**Figure 4:**
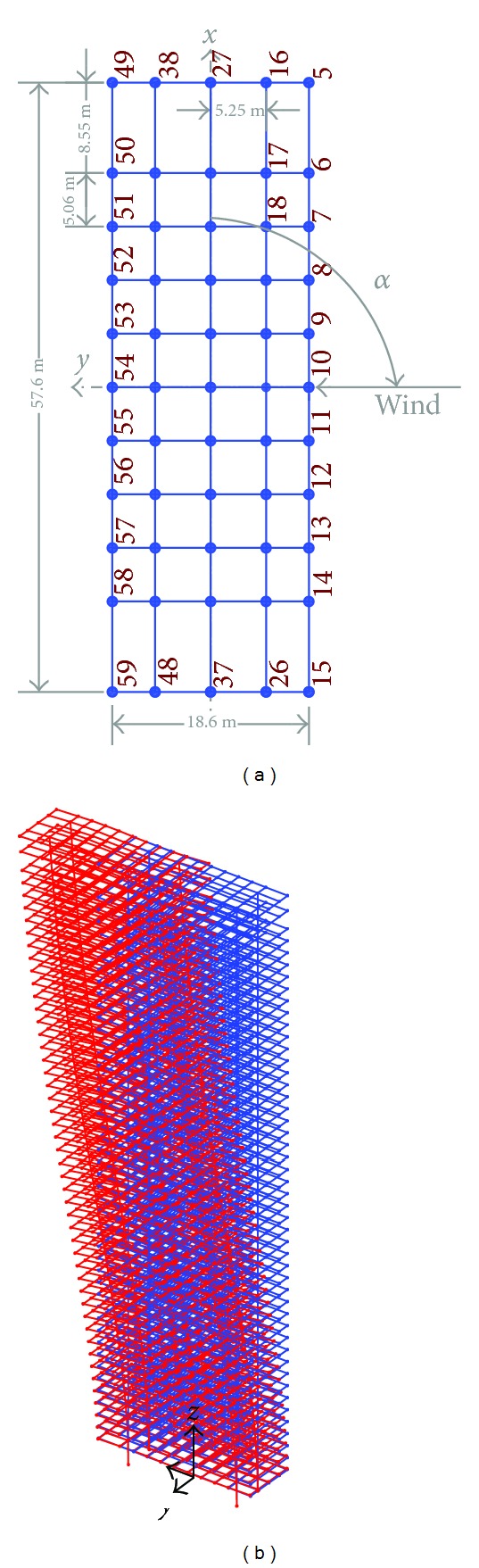
FE model of the tower with the coordinate system: (a) top view; (b) 3D view with the first modal displacements (in red). Note that the dimensions 18.6 m and 57.6 m indicate the internal steel frame and not the outer dimensions of the building.

The second step in the proposed procedure was followed to obtain an FE model of the full-scale building to be used with wind load data in order to predict the dynamic behavior in a real world scenario.  Figure 4 shows the FE model of the full-scale building tower. The model has 2644 elements; each floor has a total number of 55 elements. Two main columns to carry the vertical loads were assumed to have hollow rectangular cross sectional areas with a wall thickness varying with height in a step manner (rigidity changing with height). Floor masses were assumed to be distributed over the beams and the columns. The structural damping ratio for the first mode is 1%. The modal parameters of the FE model for the first six modes are given in Table 2. The equation of motion governing the behavior of the structure under wind loads is

(1) $$\begin{array}{c} M\ddot{X}+C\dot{X}+KX=F\left(t\right), \end{array}$$

where *M* is a mass matrix,  *X* = [*x* *y*]^*T*^ is a 2*n* × 1 vector, and *n* is the number of nodes, while *x* and *y* are vectors of displacements in the *x*- and *y*-directions; *C* is a damping matrix and *K* is a stiffness matrix. *F*(*t*) = [*F*_*x*_(*t*) *F*_*y*_(*t*)]^*T*^, in which *F*_*x*_(*t*) and *F*_*y*_(*t*) are *n* × 1 vectors of external forces acting on the nodes in *x*- and *y*-directions, respectively. Using the first nine modes given from the FE model, with the next transformation

(2) $$\begin{array}{c} X=\Phi Q, \end{array}$$

in which **F** is 2*n* × 9 matrix of eigenvectors and *Q* is 9 × 1 vector of generalized displacements; that is,

(3) Φ=[ϕ1(x1)ϕ2(x1)⋯ϕ9(x1)ϕ1(x2)ϕ2(x2)⋯ϕ9(x2)⋮⋮⋮ϕ1(xn)ϕ2(xn)⋯ϕ9(xn)ϕ1(y1)ϕ2(y1)⋯ϕ9(y1)ϕ1(y2)ϕ2(y2)⋯ϕ9(y2)⋮⋮⋮ϕ1(yn)ϕ2(yn)⋯ϕ9(yn)],  Q={q1q2⋮⋮q9}.

Substituting by (2) into (1) and premultiplying by **F** ^*T*^, one can obtain

(4) $$\begin{array}{c} \Phi^{T}M\Phi \ddot{Q}+\Phi^{T}C\Phi \dot{Q}+\Phi^{T}K\Phi Q=\Phi^{T}F\left(t\right). \end{array}$$

By assuming proportional damping, the above equation results in nine uncoupled equations

(5) $$\begin{array}{c} m_{11}{\ddot{q}}_{1}+c_{11}{\dot{q}}_{1}+k_{11}q_{2}=\overset{2n}{\underset{i=1}{\sum }}\phi_{1}\left(x_{i}\right)F_{i,t}\\ m_{22}{\ddot{q}}_{2}+c_{22}{\dot{q}}_{2}+k_{22}q_{2}=\overset{2n}{\underset{i=1}{\sum }}\phi_{2}\left(x_{i}\right)F_{i,t}\\ \vdots \\ m_{99}{\ddot{q}}_{9}+c_{99}{\dot{q}}_{9}+k_{99}q_{9}=\overset{2n}{\underset{i=1}{\sum }}\phi_{9}\left(x_{i}\right)F_{i,t}, \end{array}$$

where *m*_*ii*_, *c*_*ii*_, and *k*_*ii*_ are generalized mass, generalized damping, and generalized stiffness of mode *i*, respectively. The *q*_*j*_(*t*) are then solved from each of the above equations. A MATLAB code was written to compute the time history of the responses [39].

### 2.3. Time History of the Forces

Finally, the wind loads obtained from experimental testing were scaled up to be used in the above mentioned equation of motion. Using the measurements obtained by the pressure transducers, *C*_*p*_ at each tap was obtained as a function of both space and time. The geometric scale of the model to the prototype *λ*_*L*_ is 1 : 100. The mean wind speed at full-scale is assumed to be 30 m/s at a height of 100 m and the mean wind speed during the wind tunnel tests was roughly about 15 m/s (see Table 1). This gives a velocity scale *λ*_*U*_ of about 1 : 2. Accordingly, the time scale can be calculated as *λ*_*T*_ = *λ*_*L*_/*λ*_*U*_ = 1 : 50. This means that the 2 minute test duration corresponds to 100 minutes at full-scale. The pressure values on the surface of the full-scale model can be calculated as follows:

(6) $$\begin{array}{c} P\left(\text{space},\text{time}\right)=\frac{1}{2}\rho U^{2}C_{p}\left(\text{space},\text{time}\right), \end{array}$$

where *P*(space, time) is a matrix containing pressure values on the surface of the full-scale building as a function of space and time, *ρ* is the air density which is assumed to be 1.225 kg/m^3^, *U* is the mean velocity of the wind at full-scale, and *C*_*p*_ is the pressure coefficient obtained at the location of each tap as a function of time. The wind load at each node of the outer surface is the integration of the pressure over the surface area in the vicinity of the node (tributary area, see [40]) as follows:

(7) $$\begin{array}{c} F\left(\text{nodes},\text{time}\right)=\int P\left(\text{space},\text{time}\right)dA. \end{array}$$

Once the time history of the pressures on the outer surfaces is calculated, the external forces acting on the nodes of the surface can be computed. The excitation forces acting on the internal nodes are of course equal to zero. Codes were written in MATLAB to estimate the time histories of the wind forces acting at the external nodes of the FE model. The generalized forces (GF) are obtained as follows:

(8) $$\begin{array}{c} \text{GF}\left(t\right)=F^{T}F\left(\text{nodes},\text{time}\right). \end{array}$$

## 3. Responses under Wind Loads

By numerically integrating the ordinary differential equations in (5), one can obtain the time history of displacements, accelerations, internal loads, and base loads for the full-scale building. The numerical integration was carried out using Simiulink implemented in MATLAB [39]. To allow for better understanding the interference effects for the surrounding buildings, the design wind speed was assumed to be 30 m/s for all direction angles. The terms along-wind and cross-wind responses refer to the response of a top corner of the building in *x*- and *y*-directions. The response is called along-wind in *x*- or *y*-direction if the wind direction coincides or has a small shift angle with the axis *x* or *y*, respectively. Also, the response is called cross-wind in *x*- or *y*-direction if the wind direction coincides or has a small shift angle with the coordinate *y* or *x*, respectively. Note that both the along-wind and the cross-wind responses represent the lateral in-plane responses in addition to the torsional contribution. Figures 6–11 show displacement and acceleration responses of the top corner of the building in both *x*- and *y*-directions as a function of the wind direction angle. The legends I, II, BLa, BLb, and SF refer to the responses under wind flows and site configurations with the characteristics listed in Table 1 and plotted in Figures 2 and 3.

Figure 6 shows mean displacement responses in the *x*- and *y*-directions. The mean displacement responses in the *y*-direction are larger than those in the *x*-direction because of the geometry (different projected (drag) areas in the two directions). As expected, mean displacement responses are relatively high in the along-wind direction. The effect of a high-rise building in the upstream flow (with a height of about 80% of the main building) resulted in a reduced mean displacement response (*y*-direction in configuration II, at 90°). The turbulence intensity does not significantly influence the mean values of the displacement response when the spectra are matching at high frequencies (flow I, II, BLa, and BLb). However, the along-wind response in the *y*-direction under smooth flow (SF) is relatively reduced due to the lack of high-frequency turbulence (see Figure 3(b) at 90°). It is worth noting that high-frequency turbulence in a test flow should be matching with a target real-life spectrum in order for mean wind loads to be accurately simulated in a laboratory [25, 37, 41]. The high-frequency turbulence (small eddies) in a test flow is a key parameter that affects the flow patterns around a bluff body, and hence mean values of loading.

**Figure 5:**
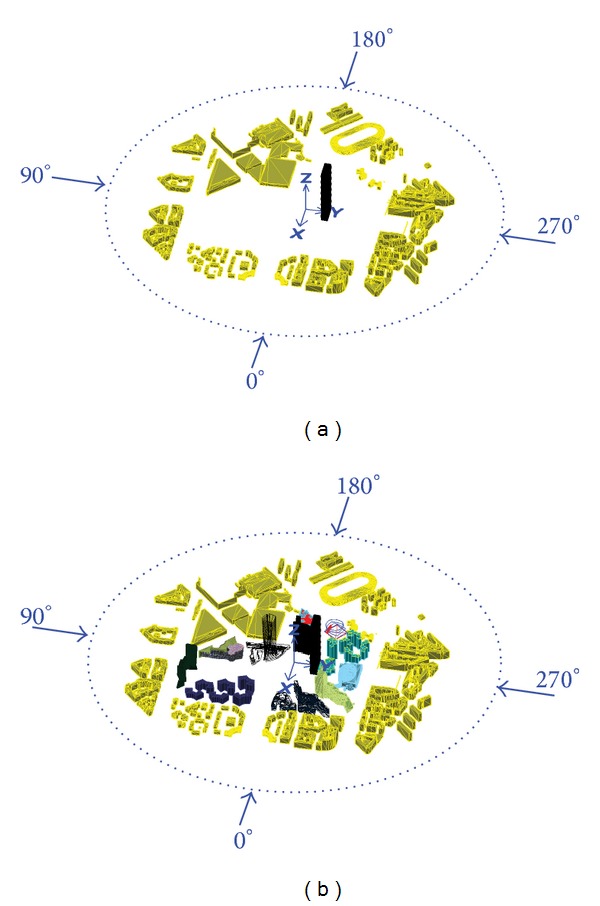
Site configurations at different wind direction angles: (a) configuration I; (b) configuration (II).

Figures 7 and 8 show standard deviation (STD) values of the displacement and the acceleration responses. The responses strongly depend on both the wind direction and the turbulence intensity. Generally, the values increase with increased flow turbulence. For the same turbulence intensity, STD responses in the cross-wind direction are larger than those in the along-wind direction. The figures also show that the effect of low-rise buildings in the surrounding (configurations I and II as shown in Figure 5) resulted in an increase in the fluctuating along-wind response (responses in the *y*-direction at 270° are higher than those at 90°) but reduction in the cross-wind response (responses in the *x*-direction at 270° are lower than those at 90°) when they are closer to the main building. This means that short buildings performed like roughness elements in the along-wind direction and increased the turbulence in the upstream flow. That is the increase in the upstream flow fluctuations may decrease the cross-wind responses at a certain level of turbulence. Moreover, by comparing the responses obtained under configurations I, BLa, BLb, and SF, one can see that the along-wind responses generally increase with the increase of turbulence. This reveal an important fact; that is, the dynamic responses in the along-wind direction increase with the increase in turbulence intensity. Aerodynamic fluctuating loads on a tall building basically result from the atmospheric turbulence and the wake excitation due to vortex shedding. The atmospheric turbulence in the approach flow causes fluctuating along-wind loading and wake excitation induces fluctuating cross-wind loads.

One can see also that all the responses (STD displacements, STD accelerations, peak displacements, and peak accelerations) in the *x*-direction under wind configurations I, II, BLa, and BLb are higher while coinciding or close to the cross-wind loads (directions 90° and 270° and their vicinities) rather than along-wind loads (angles 0°, 180°, 360°, and vicinities). This reveals the importance of the procedure followed in the current study as the codal methods provide details to calculate the along-wind response but less guidance on the evaluation of the cross-wind and torsional responses. Similar results are achieved for the responses in the cross-wind of the *y*-direction (Figures 7(b)–10(b) at 0° and 180° and their vicinities) under the five configurations except for the peak displacements that are high in the along-wind direction (Figure 9(b) at 90°, 270°, and their vicinities). The along-wind peak displacement response in the *y*-direction is higher than that in the cross-wind direction due to the fact that it depends also on the mean wind load which is high due to the rectangular cross section of the building (i.e., the drag face of the building is wider in the *y*-direction than that in the *x*-direction). However, the rest of the along-wind responses in the *y*-direction still have significantly high values (with respect to the cross-wind responses in the *y*-direction) when compared with those in the *x*-direction (cross-wind responses are much higher than along-wind responses in *x*-direction). This is because the contribution of the torsional response to the overall response in the *y*-direction is about 3.1 times the contribution of the torsional response in the *x*-direction. Frame cross sectional dimensions are 57.6 m perpendicular to the *y*-direction and 18.6 m perpendicular to the *x*-direction (see Figure 4). This means that torsional oscillations result in translations of the corner in the *y*-direction that is about 3.1 (57.6/18.6) times that in the *x*-direction.

**Figure 6:**
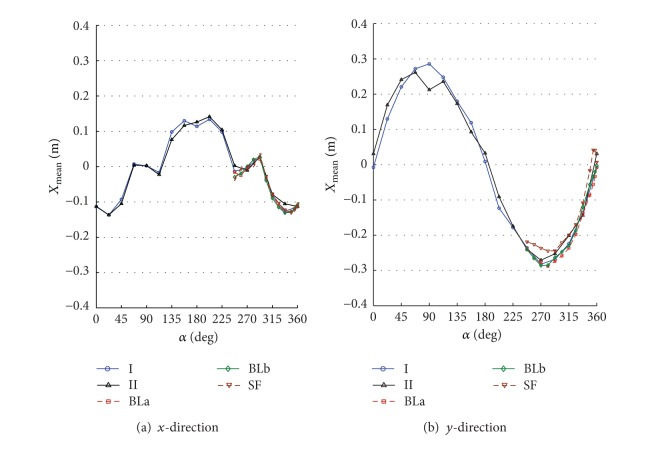
Mean displacement response of the top corner of the building under different flow conditions and site configurations: (a) *x*-direction; (b) *y*-direction. Mean responses in the *y*-direction are larger than those in the *x*-direction because of the geometry (different projected areas in the two directions). The sheltering effect from a high-rise building in the upstream flow resulted in a reduced mean displacement response (*y*-direction at 90° with configuration II). The turbulence intensity does not significantly influence the mean values of the displacement response when the high frequency parts of the wind spectra are matching (configurations I, II, BLa, and BLb as shown in Figure 3). However, the along-wind response in the *y*-direction under smooth flow (SF) is relatively reduced due to the lack of high-frequency turbulence.

Figure 9 shows that the main cause for peak cross-wind displacements in the *x*-direction at 270° is turbulence (*B*/*D* = 2.6). The smooth flow resulted in very low cross-wind peak displacements when compared with other turbulent flows. On the other hand, peak displacement response in the cross-wind direction for a prism with *B*/*D* = 0.4 is significant under both low and medium turbulence flows (configurations SF and BLb as shown in Figure 9(b) at 360°). This is due to contributions from vortex shedding as discussed in Section 4. It can be seen also that there is an angle at which the maximum values of the responses occur rather than 0°, 90°, 270°, or 360° and the shift from these angles depends on the turbulence intensity of the wind flow. The amount of shift increases for both low turbulence (SF) and high turbulence (I, II, BLa), but it is not significant for medium turbulence (BLb). The shift is also notable in the mean displacements (Figure 6), but with no dependence on the turbulence. The shift from traditional 0° and 90° is also noticed for a rectangular prism with *B*/*D* = 2 in Matsumoto et al. [42].

The interference effect of the tallest building in the surrounding, which generally has a building height of about 80% of the tower's height, resulted in a reduction in the maximum and mean along-wind displacement response in the *y*-direction when the tower was partially covered by this building (see Figure 9(b) with configuration II at angle 90°). However, this effect has no significant reduction on the other responses which mainly depend on the fluctuating component of the wind load. Figures 9 and 10 (with Figure 5(b); configuration II at 292.5°) indicate that the cross-wind maximum displacement and acceleration responses in the *x*-direction are highly increased due to the wake generated behind a building in the surrounding when located upstream (this building has a height of about 50% of the main building's height).

## 4. Discussion

### 4.1. The Influence of Turbulence

Research carried out by the author in Aly and Bitsuamlak [37], Aly et al. [24], and Fu et al. [36] shows that small structures can be tested in flows that lack large-scale turbulence. The three-second (3-s) gust peak load coefficients defined in Aly and Bitsuamlak [37] can be used to estimate peak wind loads on small structures (solar panels). By reducing the turbulence intensity, laboratory models can be tested when the objective is to estimate the 3-s peak loads, irrespective of spectral mismatch at low frequencies. However, the 3-s spectrum should be matching with full-scale; that is, high-frequency turbulence in the laboratory flow should be matching with that in nature. Accordingly, it is possible to test models in artificial wind with relatively low turbulence. It is worth noting that additional research carried out by the author [36] shows that testing of residential homes can be achieved in low turbulence flows. Such conclusions remain in force for testing rigid models where the flow structure interaction (aeroelasticity) is not an issue and the test models are relatively small in nature where the correlation in the wind flow over the test object is relatively high. These correlations are relatively high over typical residential homes [36]. Such flow can be produced in wind tunnels and new open-jet facilities with minimal efforts. However, the requirements for testing large and flexible structures at open-jet facilities are the same as for wind tunnels. That is, the mean wind velocity profile, the turbulence intensities, and scales of turbulence should be closely matched to the atmospheric boundary-layer in the desired terrain roughness [43, 44]. In any case, still the most significant challenge with open-jet tests is creating an accurate atmospheric boundary-layer, especially from a spectral perspective. The challenge is primarily caused by a relatively short test section that does not allow turbulence to be fully developed. Instead, the fans are driven actively by changing their rpm to allow for turbulence generation. By doing so, the spectral content of the flow may not by fully matched with that in nature.

Turbulence is essential for the simulation of wind loads on large and high-rise structures. The current study shows the importance of the large eddies in the flow as the mechanism by which the fluctuating structural responses are governed. In addition, the lack of small-scale turbulence may lead to an underestimation of the mean response. This is in agreement with the results published in Lee [25] who found that at high values of turbulence scale, the mean drag forces on square prisms tend to have a large constant value compared to low turbulence scales. The STD and peak responses of the structure in a smooth flow are so far from those in a turbulent flow. The spectra of the wind profiles used in the current study show that the medium-turbulence flow (BLb), which is lacking some large eddies but has sufficient high frequency content, has different response characteristics than the fully turbulent flow.

Even so, the vortex shedding noticed in the current study raises a question: *is it appropriate to test tall buildings under smooth flow?* While the two mechanisms of response are different, the smooth flow approach could be useful. The answer however requires additional research to check if the design for vortex shedding alone, provided that the lock-in range is experimentally or computationally estimated, will produce design loads similar to those from a fully turbulent flow. More research is required to investigate the possibility of testing under vortex shedding and comparing the responses to extreme responses obtained under real turbulent flows. Researchers are welcome to carry out these investigations on slender and flexible structures.

### 4.2. Vortex Shedding

Cross-wind oscillations can be excessively high, especially for slender and low damped structures. The most common source of cross-wind excitations is that associated with vortex shedding [45, 46]. Tall buildings are bluff (as opposed to streamlined) bodies that cause the flow to separate from the surface of the structure, rather than follow the body contour (Figure 11). The asymmetric pressure distribution, created by the vortices around the cross section, results in an alternating transverse force as these vortices are shed. The conditions for resonance would exist if the vortex shedding frequency becomes close to the natural frequency of the structure. This situation can give rise to very large cross-wind oscillations (even in smooth flow).

**Figure 7:**
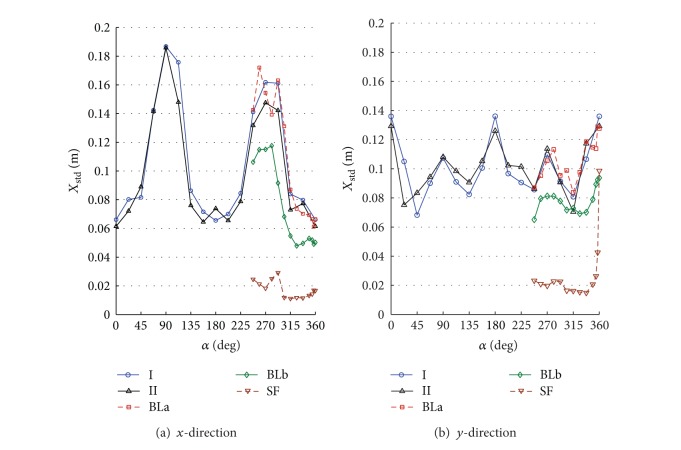
STD displacement response (*X*_std_) of the top corner of the building under different flow conditions and site configurations: (a) *x*-direction; (b) *y*-direction. Turbulence is very significant in increasing the cross-wind responses in the two directions. The smooth flow resulted in a relatively high cross-wind response in the *y*-direction (*B*/*D* = 0.4) at 360° due to vortex shedding (see Figure 11).

**Figure 8:**
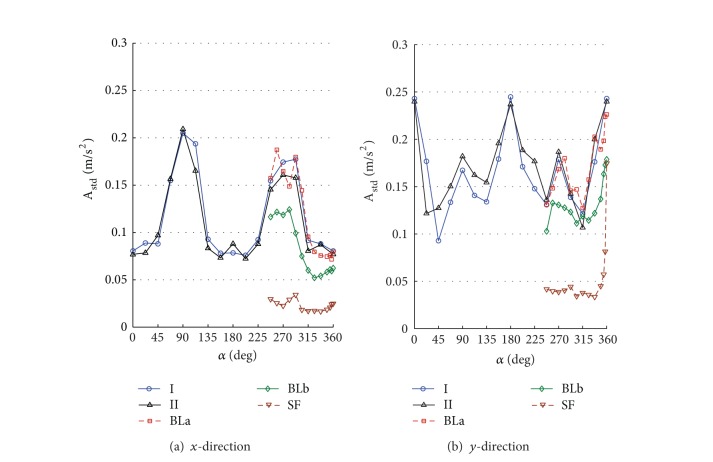
STD acceleration response (*A*_std_) of the top corner of the building under different flow conditions and site configurations: (a) *x*-direction; (b) *y*-direction. Turbulence is very significant in increasing the cross-wind responses in the two directions. The smooth flow resulted in a relatively high cross-wind response in the *y*-direction (*B*/*D* = 0.4) at 360° due to vortex shedding (see Figure 11).

**Figure 9:**
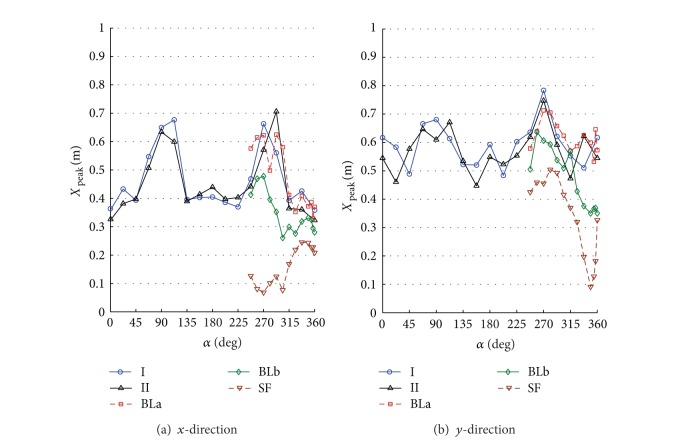
Peak displacement response of the top corner of the building under different flow conditions and site configurations: (a) *x*-direction; (b) *y*-direction.

**Figure 10:**
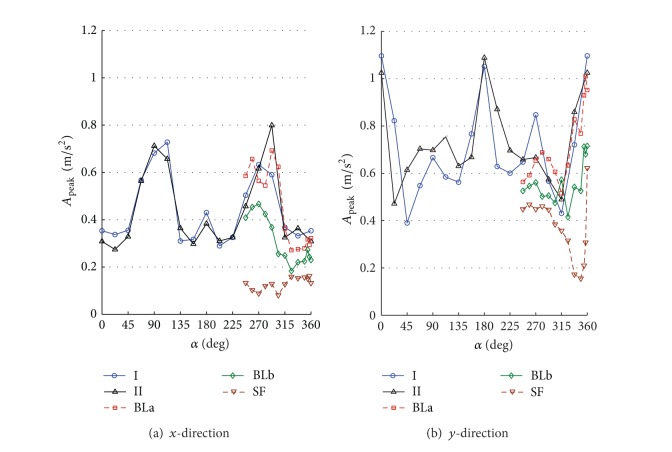
Peak acceleration response of the top corner of the building under different flow conditions and site configurations: (a) *x*-direction; (b) *y*-direction. Turbulence is very significant in increasing the cross-wind responses in the two directions.

**Figure 11:**
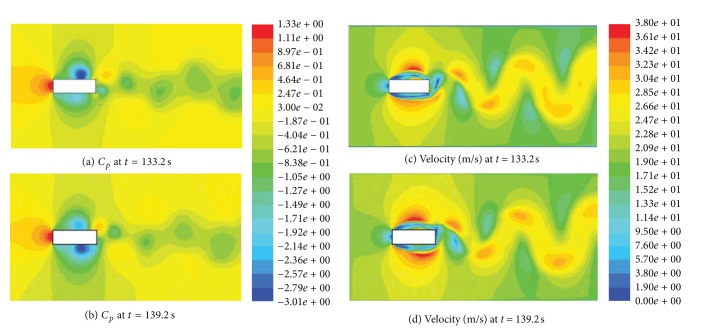
Vortex shedding phenomenon simulated in CFD: (a) (b) pressure coefficients at two instants; (c) (d) corresponding velocity contour. The shedding frequency is about 0.17 Hz, which corresponds to a Strouhal number of about 0.2. The rectangular section resulted in a relatively high response in the *y*-direction under smooth flow (*B*/*D* = 0.4).

**Figure 12:**
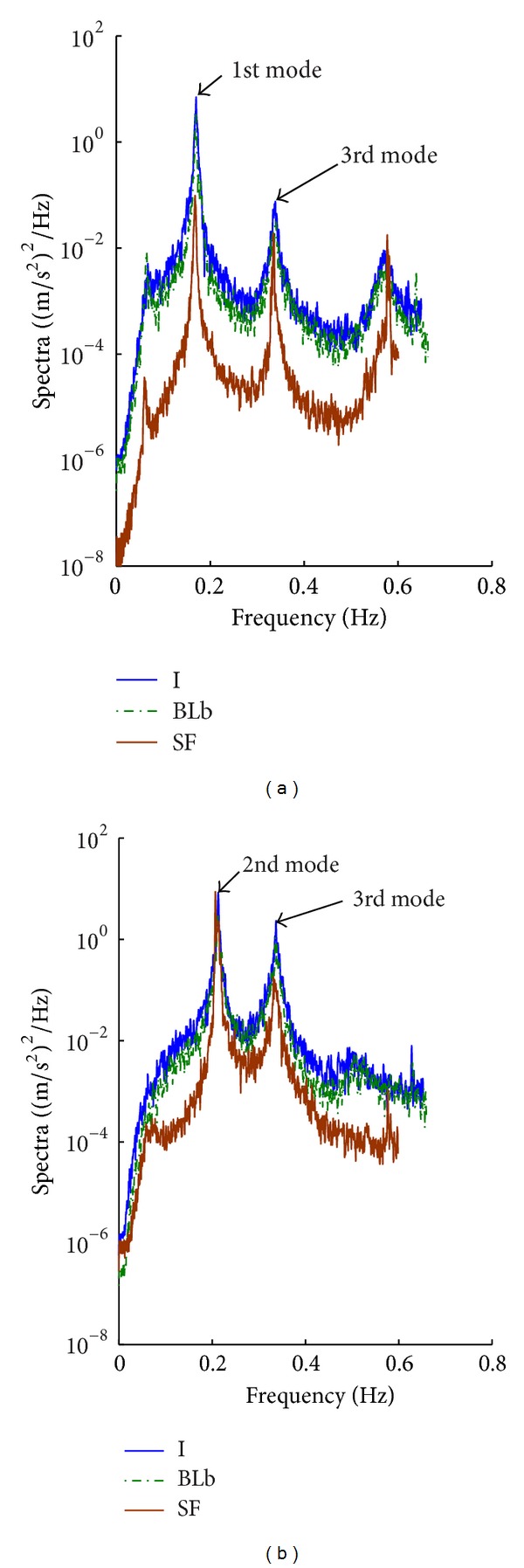
Spectral content of the cross-wind acceleration response: (a) *x*-direction at 90°; (b) *y*-direction at 360°. The turbulence effect in the *x*-direction gave a high response as a result of a shorter separation bubble and earlier flow reattachment for *B*/*D* = 2.6, compared with smooth flow. On the other hand, the rectangular section with *B*/*D* = 0.4 resulted in a high response in the *y*-direction in smooth flow as well as in turbulent flow. The high response under the smooth flow is contributed basically by vortex shedding which occurs at a frequency of about 0.17 Hz (see Figure 11), closer to the second mode of vibration (0.21 Hz) than the third mode (0.34 Hz).

The turbulence effect in the *x*-direction (Figure 12(a)) resulted in shortening the separation bubble and earlier flow reattachment for *B*/*D* = 2.6, compared to the smooth flow [18]. This could be the reason for the relatively high cross-wind response in the *x*-direction at 90° and 270° under turbulent flows, compared to smooth flow at 270° (Figures 7–10). On the other hand, the rectangular section with *B*/*D* = 0.4 resulted in relatively high responses in the *y*-direction smooth and turbulent flows (Figures 7–10 at 360°). The high response under the smooth flow comes basically from vortex shedding that occurred at a frequency close to the second mode of vibration (see Figure 12(b)). The vortex shedding phenomenon was further investigated by CFD simulations as shown in Figure 11. With ICEM CFD [47], the mesh has been created and exported as unstructured mesh (*.msh*) file that was read by FLUENT [48] which was used as a solver and for postprocessing. The Reynolds Stress Model (RSM) of turbulence was used, which reduced computational time in comparison with Large Eddy Simulations (LES). The following parameters were adopted: linear pressure-strain for the RSM, wall boundary condition from *k* equation and wall reflection effects for Reynolds-stress options, standard wall functions for near-wall treatment, *C*_mu_ equal to 0.09, *C*_1_-Epsilon equal to 1.44, *C*_2_-Epsilon equal to 1.92, and *C*_1_-PS equal to 1.8. More description of these parameters can be found in the FLUENT manual [48]. The simulations indicate that the shedding frequency is about 0.17 Hz, which corresponds to a Strouhal number of about 0.2.

### 4.3. Structural Mitigation

Wind-induced response in tall buildings depend on, among other factors, (1) terrain or mean wind velocity profile and turbulence characteristics, (2) building's aerodynamic shape, (3) wind speed, (4) wind direction angle which can be a key parameter for building's orientation change and hence reducing loads/responses, and (5) structural properties that may magnify the wind loads (resonance). As the structures become more slender and flexible, the wind-induced internal stresses and accelerations may become critical design factors. With novel design approaches, these wind-induced loads and responses could be reduced. For example, the structural engineer can control the structural properties (through member size adjustment or materials change), which affect inertial components of the wind-induced forces. Potential reduction in the inertial components of the wind loads in sensitive structures, such as tall buildings, could be achieved by “tuning” the structural properties of the building (dynamic optimization). In addition, by enhancing the inherently low structural damping, one can reduce the wind-induced loads/responses that can help achieve the most economical design. Practical solutions to increase damping in structures include the use of external dampers such as tuned liquid dampers, tuned mass dampers, viscous dampers, and smart dampers [16, 17, 49–55]. However, identifying the optimal type, location, and capacity of dampers as well as their control strategy (e.g., semiactive or active) requires additional research as each building may represent a unique case study.

**Figure 13:**
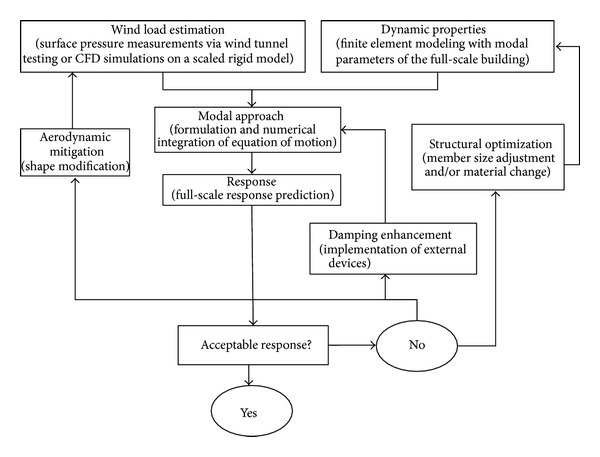
Decision making chart useful for the design of buildings for extreme wind.

The methodology presented in the current paper has the advantages of considering complex shapes of structures with nonuniform mass distribution and can easily account for different flow characteristics and any number of mode shapes to be considered in the response analysis [10]. Wind-induced response analysis of tall buildings in their preliminary design stages can help decision makers to choose among potential mitigation solutions like aerodynamic shape modification, structural member size adjustment, and/or damping enhancement by passive, active, or semiactive control devices [16, 17, 49–55].  Figure 13 shows a schematic representation of a decision making strategy, helpful in the design of high-rise buildings for wind. Structural response prediction and reduction form an integral part of the building design process, providing architects and engineers with a comprehensive understanding of the interaction between environmental factors and building design. A proper connotation of this interaction can provide significant cost savings to building owners in terms of developmental, material, and operational costs. To expose them to the methods and procedures for the efficient application of wind studies in designing a building more economically than a similar building designed with more conservative building code provisions. By doing so, the dynamic response of the building to wind effects (buffeting and vortex shedding) is virtually eliminated, leading to substantially reduced lateral design forces and assured occupant comfort. Substantial reductions in structural member size and construction cost savings can be realized in many cases. This may significantly improve the economic viability and sustainability of a development.

## 5. Conclusions

Aerodynamic loads acting on a rectangular cross-section high-rise building based on an experimental approach using a boundary-layer wind tunnel are utilized with an FE model of the building to predict its dynamic responses under wind loads. This approach has the advantages of combining along-wind, cross-wind, and torsional responses altogether simultaneously. The technique used allows for the contribution of higher modes of vibration. The paper is very rich and the flow effects on buildings are complicated by turbulence, orientation (wind direction), and the shape of the prism. The main objective of this study is to further the understanding of wind effects on tall buildings and the behavior of high-rise structures under wind conditions by means of modal analysis in the time domain and wind tunnel tests, in order to apply such knowledge to design. The main contributions of the current paper are summarized as follows.

- The case study building represents an engineered steel design of a structure that is very vulnerable to wind loads. This may be due to its low weight as well as high flexibility associated with the low dominant natural frequencies and the high aspect ratio.
- Mean displacement response is shown to be independent of the turbulence intensity, when the high frequency parts of the spectra of the test flows were matching. However, mismatch of the high frequency turbulence resulted in mismatch in the mean values of the displacement. The smooth flow, which lacks both low- and high-frequency turbulence, underestimated the mean value of the response in the along-wind direction for a prism with *B*/*D* = 2.6. On the other hand, when *B*/*D* = 0.4 the mean value did not show significant dependence on turbulence.
- The existence of short buildings in the upstream wind increases the turbulence which results in an increase in the along-wind responses but a general decrease in the cross-wind responses. The effect of the interference of the surrounding tall buildings (that generally have heights of about 81.3% of the tower) has a general effect of a reduction in the mean and the maximum along-wind displacement if the wind is coming from their direction.
- The effect of the wind direction angle is very important as the highest values of the displacement and acceleration responses may occur at angles rather than 0° or 90° (at which it is difficult to calculate such responses using traditional methods). At the same turbulence intensity, cross-wind responses are much higher than the along-wind responses for both *B*/*D* = 2.6 and *B*/*D* = 0.4. The cross-wind response is mainly contributed by both upstream turbulence and vortex shedding. The cross-wind response for *B*/*D* = 2.6 is mainly contributed by turbulence in the upstream wind, while the cross-wind response for *B*/*D* = 0.4 is mainly contributed by vortex shedding in addition to the upstream turbulence (for the full-scale velocity considered (30 m/s)).
- The variability in the test flow (spectral content) corresponds to the large variability in the response of tall buildings. The response in a smooth flow can be totally different from a fully developed turbulent flow.
- Although cross-wind responses are shown to be more significant than the along-wind responses, even under smooth flow conditions where the response is mostly contributed by vortex shedding, the increase in the inflow turbulence can increase the cross-wind response which makes testing of large and flexible structures under real turbulent flow conditions indispensable. This means that wind tunnels remain very important facilities as a technique by which a fully developed turbulent flow can be generated for testing large and flexible structures.
- The methodology followed in the current study allows for response prediction of tall buildings under real flow conditions. This is a very important step for new constructions and exiting ones, as to allow for considering mitigation strategies, including shape adjustment, structural optimization, orientation change (for new constructions), and/or addition of external damping devices, with the objective to improve the resiliency and the serviceability of buildings under extreme wind conditions.

## Figures and Tables

**Table 1:** Flow characteristics.

Configuration	Description	*I*_*u*_ (%)	*U* (m/s)
I	Tower + short buildings	22.3	14.56
II	All of the buildings	22.3	14.51
BLa	Tower alone	18.4	14.21
BLb	Tower alone	14.6	14.50
SF	Tower alone	1.8	15.63

**Table 2:** Modal parameters of the FE model.

Mode number	Generalized mass ×10^7^ (kg·m^2^)	Generalized stiffness ×10^9^ (N·m)	Frequency (Hz)	Modal damping
1	1.30	0.015	0.17	0.010
2	0.99	0.018	0.21	0.011
3	0.49	0.02	0.34	0.015
4	0.87	0.11	0.57	0.023
5	0.83	0.22	0.81	0.033
6	0.35	0.16	1.07	0.043

Modes 1 and 4 are lateral modes in the *x*-direction; modes 2 and 5 are lateral modes in the *y*-direction, while modes 3 and 6 are torsional.
